# Mouse Gut Microbiome-Encoded β-Glucuronidases Identified Using Metagenome Analysis Guided by Protein Structure

**DOI:** 10.1128/mSystems.00452-19

**Published:** 2019-08-27

**Authors:** Benjamin C. Creekmore, Josh H. Gray, William G. Walton, Kristen A. Biernat, Michael S. Little, Yongmei Xu, Jian Liu, Raad Z. Gharaibeh, Matthew R. Redinbo

**Affiliations:** a Department of Chemistry, University of North Carolina at Chapel Hill, Chapel Hill, North Carolina, USA; b Department of Biochemistry, University of North Carolina at Chapel Hill, Chapel Hill, North Carolina, USA; c Department of Microbiology, University of North Carolina at Chapel Hill, Chapel Hill, North Carolina, USA; d Integrated Program in Biological and Genome Sciences, University of North Carolina at Chapel Hill, Chapel Hill, North Carolina, USA; e Chemical Biology and Medicinal Chemistry, University of North Carolina at Chapel Hill, Chapel Hill, North Carolina, USA; f Department of Medicine, University of Florida, Gainesville, Florida, USA; University of California, San Francisco

**Keywords:** beta-glucuronidase, gut microbiome, mouse metagenomics, protein structure-function

## Abstract

Gut microbial β-glucuronidase (GUS) enzymes play important roles in drug efficacy and toxicity, intestinal carcinogenesis, and mammalian-microbial symbiosis. Recently, the first catalog of human gut GUS proteins was provided for the Human Microbiome Project stool sample database and revealed 279 unique GUS enzymes organized into six categories based on active-site structural features. Because mice represent a model biomedical research organism, here we provide an analogous catalog of mouse intestinal microbial GUS proteins—a mouse gut GUSome. Using metagenome analysis guided by protein structure, we examined 2.5 million unique proteins from a comprehensive mouse gut metagenome created from several mouse strains, providers, housing conditions, and diets. We identified 444 unique GUS proteins and organized them into six categories based on active-site features, similarly to the human GUSome analysis. GUS enzymes were encoded by the major gut microbial phyla, including *Firmicutes* (60%) and *Bacteroidetes* (21%), and there were nearly 20% for which taxonomy could not be assigned. No differences in gut microbial *gus* gene composition were observed for mice based on sex. However, mice exhibited *gus* differences based on active-site features associated with provider, location, strain, and diet. Furthermore, diet yielded the largest differences in *gus* composition. Biochemical analysis of two low-fat-associated GUS enzymes revealed that they are variable with respect to their efficacy of processing both sulfated and nonsulfated heparan nonasaccharides containing terminal glucuronides.

**IMPORTANCE** Mice are commonly employed as model organisms of mammalian disease; as such, our understanding of the compositions of their gut microbiomes is critical to appreciating how the mouse and human gastrointestinal tracts mirror one another. GUS enzymes, with importance in normal physiology and disease, are an attractive set of proteins to use for such analyses. Here we show that while the specific GUS enzymes differ at the sequence level, a core GUSome functionality appears conserved between mouse and human gastrointestinal bacteria. Mouse strain, provider, housing location, and diet exhibit distinct GUSomes and *gus* gene compositions, but sex seems not to affect the GUSome. These data provide a basis for understanding the gut microbial GUS enzymes present in commonly used laboratory mice. Further, they demonstrate the utility of metagenome analysis guided by protein structure to provide specific sets of functionally related proteins from whole-genome metagenome sequencing data.

## Abstract (precis)

Mice are commonly employed as model organisms of mammalian disease; as such, our understanding of the compositions of their gut microbiomes is critical to appreciating how the mouse and human gastrointestinal tracts mirror one another. GUS enzymes, with importance in normal physiology and disease, are an attractive set of proteins to use for such analyses. Here we show that while the specific GUS enzymes differ at the sequence level, a core GUSome functionality appears conserved between mouse and human gastrointestinal bacteria. Mouse strain, provider, housing location, and diet exhibit distinct GUSomes and *gus* gene compositions, but sex seems not to affect the GUSome. These data provide a basis for understanding the gut microbial GUS enzymes present in commonly used laboratory mice. Further, they demonstrate the utility of metagenome analysis guided by protein structure to provide specific sets of functionally related proteins from whole-genome metagenome sequencing data.

## INTRODUCTION

The bacteria in the gut play a critical role in gastrointestinal homeostasis and disease states and encode specific enzymes that directly influence human health (1, 2). For example, gut microbiome proteins involved in the processing of drugs such as digoxin, 5-fluorouracil, methotrexate, and irinotecan have been identified and their influence in therapeutic outcomes has begun to be appreciated (3–7). Similarly, endogenous and dietary compounds have also been connected to specific microbiome-encoded enzymes (8). Thus, to link the biochemistry of microbiota enzymes with mammalian physiology, it is essential to update the “one enzyme-one substrate” paradigm to the microbiome, characterizing key enzymes as well as identifying their primary substrates and products. Additionally, it is important to understand the structural and functional diversity of the microbial enzymes present in the mammalian gut.

The gut microbial β-glucuronidase (GUS) enzymes had been hypothesized to be responsible for the dose-limiting adverse outcomes caused by administration of the anticancer drug irinotecan as early as 1995 (9), and β-glucuronidase activity had been known to be present in mammalian feces since the early 1970s (10–13). Their role in irinotecan toxicity was established in 2010 and was also controlled using microbial GUS-specific inhibitors that alleviated intestinal damage and diarrhea (7). This approach has also been expanded to prevent adverse intestinal outcomes associated with nonsteroidal anti-inflammatory drugs (14). Thus, analyses of microbial GUS enzymes helped establish that the gut microbiome contains drug targets that can be selectively modulated using small-molecule inhibitors.

Given their role as drug targets, it was crucial to define the diversity of GUS enzymes in the gut microbiome. We recently presented the first atlas of GUS enzymes identified in the human gut microbiota (15). The Human Microbiome Project (HMP) samples were collected from 139 healthy donors that gave rise to 4.8 million unique gene products; using structure-guided features specific to GUS enzymes, we identified 279 distinct GUS proteins in this HMP data set—an HMP GUSome. Additionally, we categorized the HMP GUS proteins into six structural classes and demonstrated that they sampled different levels of activity with distinct glucuronic acid-containing substrates (15).

The process the we employed can be described as metagenome analysis guided by protein structure. Often, analysis of microbial metagenome data stops at the assignment of KEGG classifications, which are broad definitions of what type of protein is encoded by a family of genes (16). Such assignments, if performed correctly, would have grouped all 279 *gus* genes in the HMP as the “same gene.” However, they are not the same, as demonstrated by their wide range of lengths with additional substrate-binding modules, active-site features that sample diverse functions, and distinct subcellular localizations of the proteins within the HMP GUSome (15). Thus, by using features of known structures such as active-site architecture to probe large microbial metagenomic data sets, the approach employed here has the capability of uncovering further essential details present in microbial metagenome data to define families of enzymes at the level of granularity necessary to understand their specific functions and therefore the roles that they might play in mammalian-microbial symbiosis. In addition, this approach is both scalable and transferable, as it can be applied to other genes and proteins present in microbiome sequencing data.

Given the important role that mice play as model systems for human physiology and disease, we sought to create an atlas of microbial GUS enzymes from the mouse gut microbiome data—a mouse GUSome. Fortunately, a high-quality mouse gut whole-genome metagenome data set was provided by Xiao et al. in 2015 (17). These data were collected from eight mouse strains that had been obtained from five providers housed in six locations worldwide and fed two distinct diets (17). Using these data and metagenome analysis guided by protein structure, here we report the identification of 444 distinct GUS proteins from the 2.5 million unique proteins identified in the mouse gut metagenome data set. The structural and functional diversity of the mouse GUSome is outlined and compared to that of the HMP GUSome (15). Together, the results highlight the usefulness of employing protein structural information in identifying and defining the biochemical capacity present in the mammalian microbiome.

## RESULTS

### GUS identification in mouse gut metagenomes.

**FIG 1:**
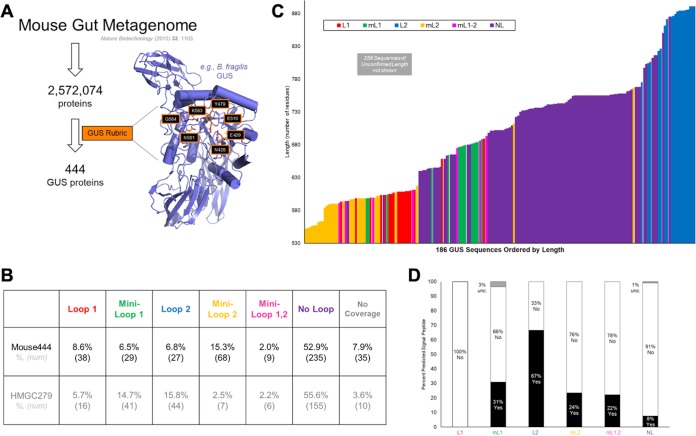
Mouse gastrointestinal microbial GUS enzyme identification, localization, and composition. (A) An assembled mouse gut metagenome data set was examined for GUS proteins using metagenome analysis by protein structure (MAPS) and following a previously outlined method (15, 17). (B) Assignment of loop classifications for the mouse GUSome (Mouse444) and previously published assignment for the human GUSome (HMGC279). “No Coverage” indicates sequences that did not have sequence information in the loop 1 or loop 2 region. (C) GUS protein length colored according to loop category. A total of 258 proteins had unclear lengths. (D) Predicted signal sequence presence classified by GUS loop category; enzymes without a clear sequence in this region are labeled “unc.” (for “uncertain”).

We used protein structure to identify GUS enzymes from the ∼2.5 million nonredundant genes in the 2015 catalog of the mouse gut metagenome (17). This metagenome was assembled from mice of both sexes housed at six distinct sites comprised of eight mouse strains from five sources and fed two diets (normal and high fat) (17). The approach employed was similar to one used previously by our group to identify the GUS proteins in the HMP stool samples (15). Briefly, the sequences of GUS enzymes of known structure (PDB code 3K46 [Escherichia coli], PDB code 4JKM [Clostridium perfringens], PDB code 4JKL [Streptococcus agalactiae], and PDB code 3CMG [Bacteroides fragilis]) were used to identify mouse gut metagenome proteins with >25% sequence identify and E values below 0.05 (though most proteins that make it through all cutoffs have E values on the order of 10^−20^) (Fig. 1A). This step selected 237,435 proteins from the 2,572,074 proteins in the mouse gut metagenome. We then selected only those proteins that maintained the active-site residues shown previously to be essential and specific to GUS proteins, including the asparagine and lysine side chains (N581 and K583 in Fig. 1A) that coordinate the carboxylic acid moiety unique to glucuronide acid relative to glucose and galactose, for example. This second step identified a set of 444 unique proteins (see Table S1 in the supplemental material) that compose a mouse intestinal “GUSome” that is the subject of our subsequent analysis. Interestingly, the 444 GUS proteins represent a value similar in magnitude to the 279 identified previously from the HMP.

10.1128/mSystems.00452-19.6 TABLE S1 Full amino acid sequences of all 444 distinct murine gut bacterial β-glucuronidases. Download Table S1, XLSX file, 0.2 MB. Copyright © 2019 Creekmore et al. 2019 Creekmore et al. This content is distributed under the terms of the Creative Commons Attribution 4.0 International license.

To fully elucidate all potential GUS enzymes in the mouse data set, we performed an iterative search that used the GUS enzymes captured using our rubric to seed another search. For example, the first round of the iterative search used the 444 enzymes discovered using enzymes of known structure (E. coli, C. perfringens, S. agalactiae, and B. fragilis) to find an additional 13 enzymes (see Fig. S1 in the supplemental material). We continued to seed future searches with newly discovered enzymes until no new enzymes were discovered in the fifth round of the search. This search left us with a total of 28 additional enzymes. However, we chose not to fully characterize these additional 28 enzymes because the sequences used to seed their discovery have not been structurally confirmed to be GUS enzymes.

10.1128/mSystems.00452-19.1 FIG S1 Number of putative GUS sequences found in each round of the iterative search using putative GUS enzymes as seeds for GUS identification. Download FIG S1, PDF file, 0.02 MB. Copyright © 2019 Creekmore et al. 2019 Creekmore et al. This content is distributed under the terms of the Creative Commons Attribution 4.0 International license.

### Mouse GUSome structural categories and subcellular localizations were compared to those of the human GUSome.

We categorized the microbial GUS proteins identified in the mouse gut into six separate loop classes given their active-site architecture using multiple-sequence alignment (MSA). We have previously shown that two loop regions at the microbial GUS active site change the substrate utilization characteristics of GUS enzymes and provide a useful method with which to group enzymes by structural features (15). In the HMP study, we termed GUS proteins loop 1 (L1) proteins, loop 2 (L2) proteins, or no-loop proteins, depending on the presence or absence of sequence insertions at positions 356 and 416 of the E. coli GUS in the conserved GUS glycoside hydrolase family 2 (GH2) fold (15). Three additional categories, namely, mini-loop 1, mini-loop 2, and mini-loop 1,2, were specified for enzymes with shorter insertions at these positions (15). A small number of proteins from this metagenomic analysis lacked sequence coverage in these regions thus were categorized as “no coverage” proteins (15).

The mouse gut GUSome also populates these categories but exhibits some features that are distinct from those of the HMP (Fig. 1B). Like the HMP279 data set, composed of the 279 unique GUS proteins identified in the Human Microbiome Project stool sample data set, the Mouse444 data set classifies the vast majority of its GUS proteins in the no-loop category. However, the Mouse444 data set exhibits more proteins in the loop 1 category (8.6%) than the human data set (5.7%) but fewer than the human set in all other loop structure categories, except the mini-loop 2 category (Fig. 1B; see also Table S2).

10.1128/mSystems.00452-19.7 TABLE S2 Analysis of murine gut bacterial β-glucuronidases. Data include loop classification, sequences of loop regions, taxonomic assignment, protein length, and signal sequence (if applicable) for each of the 444 β-glucuronidases identified. Download Table S2, XLSX file, 0.05 MB. Copyright © 2019 Creekmore et al. 2019 Creekmore et al. This content is distributed under the terms of the Creative Commons Attribution 4.0 International license.

The mini-loop 2 category shows a high proportion of proteins in mice compared to the human data set. Many of the proteins that populate this mini-loop 2 category have 8 residues in the loop 2 region, placing them on the border between the mini-loop 2 and no-loop categories and thus potentially explaining the increase compared to the HMGC279 data set. We propose that similar proteins may have been classified in the no-loop category based on the HMGC279 data set, as the lengths of most of these mini-loop 2 proteins fall in a region previously occupied by the no-loop proteins (Fig. 1C). This ambiguity with respect to edge cases highlights variability in alignment-based classification. Future functional data need to be obtained to discern how this subset of mini-loop 2/no-loop proteins should be classified. However, generally, the mouse gut GUSome samples the complete set of structural categories but contains more mini-loop 2 and loop 1 proteins than the human gut GUSome at the expense of the other categories.

Among the 444 unique GUS proteins identified here, 258 had uncertain starting methionines and were thus termed “unclear” with respect to length analysis. The 186 proteins of confirmed length from the Mouse444 data set range in length from 552 to 891 residues, with loop types largely clustered together (Fig. 1C). For example, 16 of the 18 loop 1 proteins of confirmed length are between 593 and 611 residues in length, with two proteins located outside that range. Similar trends are observed for the loop 2 proteins, which are the largest and are nearly universally greater than 800 amino acids in length (Fig. 1C). We also examined each protein for a potential signal peptide, which would be indicative of potential trafficking of the protein outside the microbial cell. A fraction of the proteins in each loop category were labeled “uncertain” because of missing or miscalled methionines or because of the presence of a cysteine immediately following the predicted peptidase I site. For the proteins assigned with confidence, we found that no loop 1 GUS proteins contained signal sequences, while approximately 8% to 67% of the other categories had signal sequences (Fig. 1D). Thus, we conclude that a significant fraction of the GUS proteins in the mouse gut microbiome can be exported extracellularly whereas the loop 1 proteins appear to remain intracellular. Similar trends were observed for the human GUSome HMP279 data set (15).

### The mouse GUSome and the human GUSome are distinct but share similar clustered characters.

**FIG 2:**
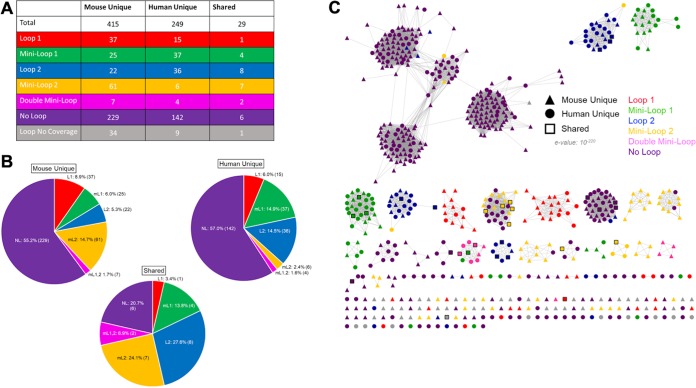
Mouse444 and HMGC279 loop classification and sequence similarity network. (A) Loop classification of numbers of proteins unique to mouse, unique to human, and shared by the two data sets. (B) Loop composition of unique mouse, unique human, and shared proteins with number and percentage of GUS enzymes for each loop category. (C) Sequence similarity network of the Mouse444 and HMGC279 data sets with loop category and source data set labeled.

Only 29 of the GUS proteins identified in the human and mouse gut GUSomes are nearly identical to one another, as defined by protein sequences that share >98% identity at the amino acid level (Fig. 2A). As such, there are 693 different proteins in these data sets, and these distinct sequences are observed for each of the structural categories of the GUSome (loop 1, etc.; Fig. 2A). Similar observations were noted when the overall gut metagenomes of mice and humans were compared and were found to contain only 102,830 identical genes among 4.1 million in human feces (0.02%) and 2.5 million in mouse fecal material (0.04%) (17). As expected, categories overrepresented in the Mouse444 data set compared to the HMGC279 data set contained more GUS proteins of unique sequence in the Mouse444 data set, such as the 37 and 61 loop 1 and mini-loop 2 proteins, respectively, unique to the mouse GUSome. In contrast, other categories contain less GUS proteins of distinct sequence, such as the 25 and 22 mini-loop 1 and loop 2 proteins, respectively, unique to the mouse GUSome (Fig. 2A and B). However, despite these differences at the amino acid level, clustering by sequence similarity networks (SSNs) revealed that the mouse GUSome and the human GUSome create highly intertwined “subnetworks” of GUS proteins (Fig. 2C). The mouse and human proteins cluster together into groups containing proteins from both GUSomes, with only a small number of multi-GUS clades containing only mouse proteins and no multi-GUS clades containing only human proteins (Fig. 2C). Similarly, in spite of their stark differences in gene composition, it was shown previously that 88% of KEGG pathways are shared between the human and mouse gut metagenomes (17). Thus, it would appear that, despite the differences in the GUS proteins in the mouse and human gut at the amino acid sequence level, the GUSomes of each mammal may converge on a shared overall functional capacity.

### Mouse GUSome phylogeny compared to human GUSome.

**FIG 3:**
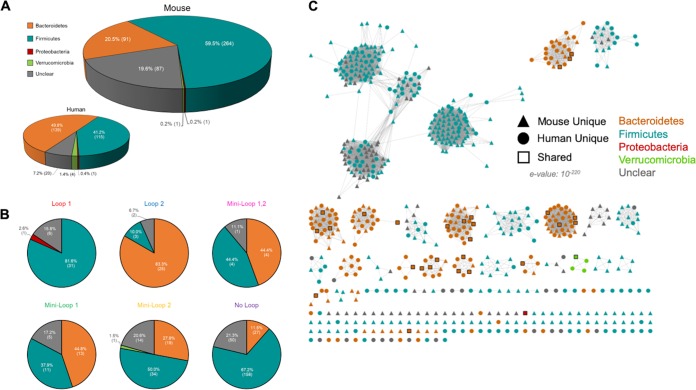
Mouse444 taxonomy and Mouse444 loop classification taxonomy. (A) Phylum composition of the mouse GUSome compared to the human GUSome with number and percentage of GUS enzymes for each phylum indicated. (B) Phylum composition of each loop category for the mouse GUSome with number and percentage of GUS enzymes for each phylum. (C) Sequence similarity network of the Mouse444 and HMGC279 data sets with phylum and source data set labeled.

The taxonomy of each protein in the Mouse444 data set was assigned, to the degree possible, via NCBI BLASTP using the nr database (18) (Fig. 3A; see also Table S2). We acknowledge that while this method does not account for horizontal gene transfer or overall microbial community change, it can provide a general sense of the phylogenetic representation of the GUSome that does change with the microbial community. We found that nearly 81% (357 proteins) could be assigned a phylum, while ∼20% (87 proteins) had unclear phylogeny (defined as either no significant similarity or a high level of similarity to more than one microbial phylum). Approximately 60% of the GUS proteins in the mouse GUSome arose from *Firmicutes* bacteria and 20.5% from *Bacteroidetes*, with a single protein from each of the *Verrucomicrobia* and *Proteobacteria* phyla (Fig. 3A). In contrast, the human GUSome HMP279 exhibited 50% *Bacteroidetes* and only 40% *Firmicutes* (Fig. 3A). While this may simply reflect a change in community structure, this observation stands in contrast to the overall similarity in taxa in the mouse and human gut metagenomes (17) and, as outlined below, may reflect differences in diet between captive mice and humans free to make dietary choices. The preponderance of *Firmicutes* in the mouse GUSome was largely reflected in the no-loop, mini-loop 2, and loop 1 enzymes, of which 67%, 50%, and 82%, respectively, were from this phylum (Fig. 3B), whereas only 50% of the no-loop proteins were from *Firmicutes* in the HMGC279. In the HMGC279, 87% of loop 1 and 64% of mini-loop 2 were *Firmicutes*, but these categories made up a smaller percentage of the total; thus, the increase in their proportion increased the *Firmicutes* proportion overall. The same sequence similarity network in Fig. 2, colored by phylum instead of by loop classification, reveals that the phyla clustered together in their own clades, similarly to the loop type data (Fig. 3C). This similar grouping indicates that, generally, loop types of similar sequences cluster within the same phylum. A similar arrangement of grouped GUS loop categories and taxonomy (e.g., *Firmicutes* loop 2 proteins) was also observed in the human GUSome.

### Strain and diet impact mouse GUSome.

**FIG 4:**
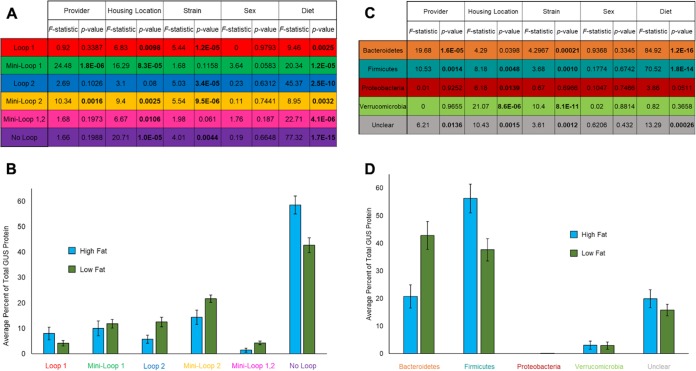
Metagenomic variable assessment and *gus* changes in diet. (A) *F-*statistic and *P* values from five-way ANOVA comparing loop category with provider, housing location, strain, sex, and diet categories. Significance was determined using the Benjamini-Hochberg method with a false-discovery rate of 0.05. (B) Percentages of total GUS composition for loop categories under conditions of high-fat and low-fat diets. (C) *F-*statistic and *P* values from five-way ANOVA comparing phylum with provider, housing location, strain, sex, and diet categories. Significance was determined using the Benjamini-Hochberg method with a false-discovery rate of 0.05. (D) Percentages of total GUS composition for phyla under conditions of high-fat and low-fat diets.

We next examined the GUS structural loop categories (e.g., loop 1, loop 2, no loop, etc.) and the phylogeny differences that might exist between the distinct variables captured in the mouse gut metagenome data set. The variables present in creating this metagenome data set were mouse strain, housing location, provider, sex, and diet. Surprisingly, we found no differences in either GUS feature (loop category distribution or phylogeny) associated with sex (Fig. 4A and B; see also Fig. S2 and Fig. S3). We had multiple significant findings across loop categories and metagenome variables (Fig. 4A; see also Fig. S2). However, we noted two interactions whose results were associated with particularly low *P* values: diet with loop 2 and diet with no loop. These *P* values were lower than the next lowest *P* value by factors of 10^4^ and 10^9^, respectively (Fig. 4A). Further evaluation of the directional difference showed that the no-loop levels increased in high-fat-diet mice and that loop 2 levels decreased in high-fat-diet mice (Fig. 4B). We hypothesize that the difference in macromolecules with glucuronides in the two diets selects for GUS enzymes (i.e., loop 2 and no loop) that can process large molecules with their relatively open active site. Similarly, analyzing differences between phylogenies and between metagenome variables, we found several significant associations (Fig. 4C; see also Fig. S3). The results seen with *Verrucomicrobia* had a low *P* value associated with strain differences (Fig. 4C). This association was due to the presence or absence of microbes of this phylum within mouse strains (Fig. S3). Again, however, two associations within the diet data stood out as having particularly low *P* values: *Bacteroidetes* and *Firmicutes* (Fig. 4C). *Firmicutes* levels increased in high-fat-diet mice, while *Bacteroidetes* levels decreased in high-fat-diet mice (Fig. 4D). This trend is likely connected to the changes in the loop 2 and no-loop categories, as the no-loop data predominantly represented *Firmicutes* and the loop 2 data predominantly represented *Bacteroidetes* (Fig. 3B); thus, the phylogenetic trend followed the loop trend. Our data show that diet effects GUS composition, which we predict is due to the different metabolic niches of GUS proteins.

10.1128/mSystems.00452-19.2 FIG S2 Percentages of total GUS composition for the indicated loop categories. (A) Strain. (B) Location. (C) Provider. (D) Sex. Download FIG S2, PDF file, 0.03 MB. Copyright © 2019 Creekmore et al. 2019 Creekmore et al. This content is distributed under the terms of the Creative Commons Attribution 4.0 International license.

10.1128/mSystems.00452-19.3 FIG S3 Percentages of total GUS composition for the indicated phylogeny categories. (A) Strain. (B) Location. (C) Provider. (D) Sex. Download FIG S3, PDF file, 0.02 MB. Copyright © 2019 Creekmore et al. 2019 Creekmore et al. This content is distributed under the terms of the Creative Commons Attribution 4.0 International license.

### The structure and function of a GUS unique to mice on a low-fat diet were characterized biochemically.

Finally, we selected two GUS enzymes associated with mice on a low-fat diet, identified the complete gene, overexpressed and purified the enzyme, and performed biochemical characterization. We chose a GUS (identifier [ID]: ref269) that was initially a fragment that was only 534 residues in length and classified as loop 2 (L2). We found using BLASTP (18) that this fragment corresponded to an 864-residue full-length protein from Bacteroides ovatus (*Bo*GUS L2).

**FIG 5:**
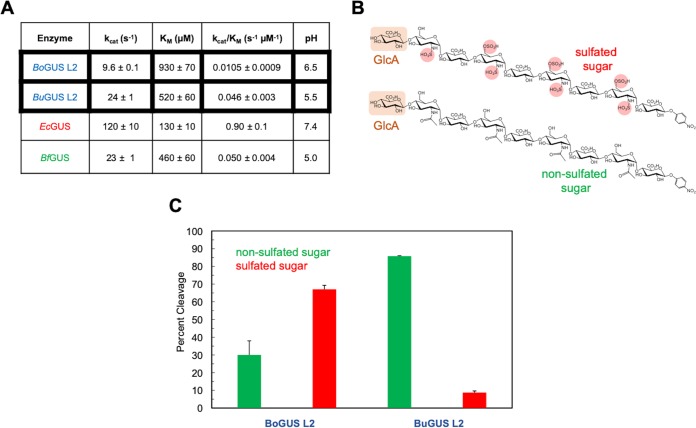
*In vitro* analysis of low-fat-diet-specific GUS enzyme *Bo*GUS L2. (A) Kinetic parameters (*k*_cat_, *K_m_*, *k*_cat_/*K_m_*) of previously uncharacterized *Bo*GUS L2, *Bu*GUS L2, and Bacteroides fragilis mini-loop 1 (*Bf*GUS mL1) with the previously published E. coli GUS L1 (*Ec*GUS L1) (7). (B) Structure of two heparan sulfate nonasaccharides tested with *Bu*GUS L2 and *Bo*GUS L2. (C) Data from testing carbohydrate glucuronides with *Bu*GUS L2 and *Bo*GUS L2.

We synthesized the gene for *Bo*GUS L2, overexpressed the protein in E. coli, and purified it using affinity and sizing column chromatography methods identical to those employed previously (15). Pure *Bo*GUS L2 exhibited its highest activity at pH 6.5 to 7.0 in the presence of the standard GUS assay reagent *p*-nitrophenyl-glucuronide (PNPG) as a substrate (Fig. S4). We then examined the kinetics of PNPG cleavage by *Bo*GUS L2 at pH 6.5 (Fig. 5A). *Bo*GUS L2 exhibited a low *k*_cat_ level and a weak *K_m_* response, resulting in a poor *k*_cat_/*K_m_* result, which indicates that this enzyme did not efficiently process PNPG. In contrast, some loop 1 enzymes, such as the E. coli loop 1 protein, exhibited better activity with PNPG whereas the B. fragilis mini-loop 1 enzyme showed poor activity akin to that seen with *Bo*GUS L2 (Fig. 5A). These results highlight the range of catalytic properties within the GUS family of enzymes.

10.1128/mSystems.00452-19.4 FIG S4 pH screen of Bacteroides ovatus L2 GUS ranging from pH 4.0 to pH 7.4 to determine the optimal pH. The optimal pH was determined to be 6.5. Download FIG S4, PDF file, 0.04 MB. Copyright © 2019 Creekmore et al. 2019 Creekmore et al. This content is distributed under the terms of the Creative Commons Attribution 4.0 International license.

We also examined the ability of *Bo*GUS L2 to process larger polysaccharide substrates. We showed previously that loop 1 GUS proteins do not process these substrates, while the more open active sites of other types of GUS, including mini-loop 1, loop 2, and mini-loop 2, are able to efficiently act on carbohydrates (15). We chose two heparan sulfate nonasaccharides (9-mers) with terminal glucuronic acid moieties at the nonreducing ends, including one with nonsulfated 9-mer and one with sulfated 9-mer (Fig. 5B). We found that *Bo*GUS L2 and Bacteroides uniformis loop 2 GUS (*Bu*GUS L2) were functionally distinct by the use of these two 9-mer substrates. *Bo*GUS L2 was less active than *Bu*GUS L2 using the nonsulfated 9-mer (30% cleavage versus 90% cleavage, respectively; Fig. 5C). In contrast, *Bo*GUS L2 efficiently processed the sulfated polysaccharide whereas *Bu*GUS L2 exhibited almost no activity with this anionic substrate (70% cleavage versus 8% cleavage, respectively; Fig. 5C). As shown by modeling performed with Phyre2 using *Bu*GUS L2 as a template (19), *Bo*GUS L2 contains four positively charged residues, including three arginine residues and one lysine residue, in place of neutral or negatively charged residues in *Bu*GUS L2 (Fig. S5), possibly explaining the ability of the active site of *Bo*GUS L2 to process the negatively charged sulfated 9-mer substrate. However, mutagenesis of these differing residues did not yield the expected change in substrate processing activity in either enzyme, demonstrating the limitations of using models (e.g., of *Bo*GUS L2) rather than complete experimental structures in guiding conclusions based on activity. Future studies will be required to determine the structural basis of the differences in sugar processing between *Bu*GUS L2 and *Bo*GUS L2. Taken together, these results highlight the importance of the use of experimental structures to unravel the molecular basis for differential substrate processing by specific gut microbial enzymes.

10.1128/mSystems.00452-19.5 FIG S5 Structure of *Bu*GUS L2 active site with select corresponding *Bo*GUS L2 residues replacing *Bu*GUS L2 residues. The *Bo*GUS L2 structure was modeled on that of *Bu*GUS L2 using the method described in reference 19. Download FIG S5, PDF file, 1.4 MB. Copyright © 2019 Creekmore et al. 2019 Creekmore et al. This content is distributed under the terms of the Creative Commons Attribution 4.0 International license.

## DISCUSSION

Given the relevance of the mouse as a model organism, we sought to understand the diversity of the members of a family of mouse gut microbiome-encoded enzymes important to the mammalian response to cancer chemotherapy and other drugs. Building on our previous work establishing a comprehensive atlas of gut microbial beta-glucuronidase (GUS) enzymes from the fecal samples in the Human Microbiome Project (15), we applied the same methods to the comprehensive mouse gut metagenome data set established by Xiao and colleagues (17). The work flow employs both general structural features present in amino acid sequence identity as well as detailed active-site residues specific to GUS enzymes (Fig. 1A). Similarly to the 279 unique GUS proteins in the HMP (human GUSome), we found 444 unique GUS enzyme orthologs in the assembled mouse gut microbial metagenome data (mouse GUSome; Fig. 1B). In addition, akin to the human GUSome, the mouse GUSome samples the same six structure-function categories of enzymes, albeit at distinct relative levels. The mice examined contained more loop 1, mini-loop 2, and no-loop enzymes and fewer of the other categories (mini-loop 1, loop 2, and mini-loop 1,2; Fig. 1B). Extending this trend, we found that the potential intracellular versus extracellular localizations were distinct between the human and mouse GUSomes (Fig. 1D). In the human GUSome, all L2, mini-loop 2, and mini-loop 1,2 enzymes contained a predicted signal sequence, indicating their potential for extracellular secretion at least to the periplasm and perhaps as soluble extracellular proteins (15). In the mouse GUSome, 33% to 78% of the enzymes in these three categories lack these sequences, indicating that these enzymes function within the bacterial cells that express them (Fig. 1D). The reasons behind these differences between human and mouse in these first GUSome catalogs are not clear are likely related to differences in diet between humans and captive mice, as discussed below.

Despite these differences, and despite the fact that only 29 GUS proteins sharing >98% sequence identity were found in both the mouse and human GUSomes (Fig. 2A), sequence similarity networks (SSNs) indicated that the 444 mouse GUS and 279 human GUS significantly overlapped (Fig. 2C). The mouse and human GUS proteins shuffle together into clades suggestive of shared structural and functional features. As such, the functional propensities of the two GUSomes to process dietary, endogenous, and xenobiotic glucuronides are likely similar at one functional level, indicated by the same general number of GUS proteins binned into similar categories and SSN clades, but are likely distinct at another, indicated by differing relative fractions in the six active-site categories and different potential subcellular localizations. While these data give us an initial layer of granularity about microbial GUS proteins in mammalian systems, they just begin to inform us about the range of functional capabilities present in humans and mouse models.

The multiple differences in the mice sampled for the gut metagenome appear to significantly affect both loop and phylogeny in variety of ways. We found *P* values much lower than other significant categories with loop 2 and no-loop associations with diet. As well, we found a similar trend with *Bacteroidetes* and *Firmicutes* and diet. These results lead us conclude loop 2 and no-loop enzymes have metabolic roles that are favored by the low-fat diet and high-fat diet, respectively. Because loop 2 is overrepresented in the low-fat diet, we chose to express and purify a L2 GUS that was initially a fragment. Via BLAST (18), we found that this sequence corresponded to Bacteroides ovatus (*Bo*GUS L2). We further showed that it exhibited distinct functional characteristics on charged and uncharged polysaccharide substrates in *in vitro* assays compared to *Bu*GUS L2 as characterized previously (15). By structural modeling, we found that *Bo*GUS L2 contains positively charged residues not present in the *Bu*GUS L2 active site. Mutagenesis at these sites did not yield the expected change activity; thus, the distinctions in processing activities may have been due to structural differences between the proteins that are not evident in the model of *Bo*GUS L2 employed. This result highlights the importance of the use of experimental structural determinations, when possible, instead of relying on protein models. Even within a single loop category associated with diet, we saw a variety of functions, highlighting protein metabolic niches even within classifications, not just between classifications. These results also indicate that the functional specialization present in the gut microbial GUSome is driven by unique active-site features that can be understood using crystal structures or structural models.

Finally, we sought to understand why mice on the low-fat diet might encode a unique set of GUS enzymes that feature the L2 active-site architecture. Normal, low-fat mouse chow is composed of roughly twice the amount of carbohydrates as high-fat chow, in which the carbohydrates are replaced with fats. Specifically, there is more sucrose, corn starch, and maltodextrin in low-fat chow than in high-fat chow (20–25). Thus, we speculate that increased levels of these carbohydrates, particularly of the polysaccharide corn starch, in the low-fat diet maintain or facilitate the growth of microbes that contain polysaccharide-processing enzymes, including loop 2 enzymes. Importantly, however, the dietary factors that are increased in the low-fat mouse chow do not themselves contain glucuronic acid. Therefore, the presence of GUS enzymes unique to the intestinal microbiota of mice on low-fat diets may be a “passenger effect” in which the *gus* genes are not required to process these dietary substrates but other gene products in the host bacterial organisms are. Alternatively, the low-fat diet may change other aspects of mammalian or microbial metabolism to increase levels of glucuronide-containing substrates in the lumen of the gastrointestinal tract. Future studies will be required to pinpoint the roles GUS enzymes play in processing dietary and nondietary substrates. These results highlight the capabilities and limitations of utilizing protein structural features to identify enzymes involved in the metabolic capabilities of the gut microbiome.

### Conclusions.

Because mice are commonly used to model human physiology and disease, it is essential to understand the functional details of how the murine gut microbiome compares to the human gut microbiome. Gut bacterial GUS enzymes are involved in responses to therapeutics and in processing a range of endobiotic glucuronides (7, 9, 26). Our results highlight the protein-level differences of the 444 and 279 GUS enzymes present in the gastrointestinal tracts of mice and humans, respectively. While only 29 proteins are identical between the two sets, the full range of GUS proteins share similar organizations into six structural classes and significantly overlap on a sequence similarity network. We found an array of differences in GUS levels and types present in mice from different providers, housed in different locations, of different strains, and on a high-fat diet. However, we found no differences based on sex.

## MATERIALS AND METHODS

### Mouse whole-genome metagenome gut data.

The mouse protein data set was obtained from a previous study (27) under ID 68678, and metagenomic reads were downloaded from the European Nucleotide Archive (study ID PRJEB7759) as FASTQ files and used for subsequent analyses (17). All original genomic data employed were from reference 17. All data generated or analyzed during this study are included in reference 17 or in this published article (and in its supplemental material).

### Mouse β-glucuronidase identification.

Raw FASTQ files from reference 27 were run through a custom, modified MOCAT2 pipeline identical to the original MOCAT2 pipeline with the exception that it assembled every sample with both the SPAdes and IDBA *de* novo assemblers (the original MOCAT2 pipeline uses only SPAdes) to generate a list of predicted protein sequences for each mouse gut metagenome. From these predicted protein lists, putative β-glucuronidase (GUS) proteins were classified as sequences that met both of two criteria: (i) >25% identity and E value of <0.05 corresponding to at least one of the four GUS-defining microbial GUS enzymes—from E. coli, B. fragilis, C. perfringens, and S. agalactiae—and (ii) alignment with at least one of these same four GUS-defining microbial GUS enzymes at all of the GUS-specific active-site residues (the NxKG motif, the catalytic E amino acids, and N and Y motifs, as described previously [15]). The putative GUS sequences found in this initial step, rather than the four GUS-defining microbial GUS enzymes, were then used to seed an additional search through the predicted proteins in an iterative manner. Rounds of searches performed in this manner continued until no more putative GUS sequences (as defined above) were extracted from the collection of all predicted proteins. All data and information about HMP GUS sequences was taken from reference 15.

### Mouse β-glucuronidase loop classification.

The mouse GUS proteins identified were subjected to multiple-sequence alignment (MSA) using Clustal Omega version 1.2.4 (28) along with GUS sequences from the following selected model organisms: Bacteroides uniformis (NCBI accession no. WP_035447612) and Faecalibacterium prausnitzii (NCBI accession no. WP_005931592). The MSA was examined for the B. uniformis loop 2 region and the F. prausnitzii loop 1 region. GUS proteins were then categorized based on sequence differences in a highly variable loop region as described previously (15). This was accomplished with a script written in Perl using the BioPerl package and BL2Seq that is available upon request.

### Sequence similarity network.

The human and mouse sequences were uploaded to the Enzyme Function Initiative—Enzyme Similarity Tool (EFI-EST; University of Illinois) website (https://efi.igb.illinois.edu/efi-est/) in FASTA format using default options for Option C (no FASTA header reading with user supplied FASTA file). Edges were declared at an E value at 10^−220^. This value was chosen to minimize the number of clusters with single members, while retaining resolution between large clusters.

### Taxonomic assignments of GUS sequences.

The nonredundant protein sequences database (nr) was searched for sequences matching protein sequences from the Mouse444 data set using NCBI BLASTP on the nr database (18). For sequences which had a top (ranked by score) BLASTp sequence of ≥95% identity, the taxonomy of the query sequence was defined as the most specific taxonomy among the sequences of ≥95% identity. For all other sequences, the top 5 BLASTp results were analyzed for agreement in taxonomy at the most specific level and were then assigned to that level if there was a consensus among all 5. If there was no consensus at any level, the protein was marked as having had an “unclear” analysis result. Data provided for the Mouse444 sequences were from the NCBI Protein Database as of May 2017 (29).

### Signal peptide identification.

The 444 mouse sequences were analyzed for the presence of signal peptides using the online LipoP 1.0 server (30). Some enzymes were classified as “uncertain” in the N-terminal region because they had missing or miscalled starting methionines and thus were not considered for potential signal peptide presence.

### Statistical analysis of metagenome variables with loop and phylogeny.

MATLAB (Mathworks) was used to perform all statistical analysis. Significance was determined by using five-way analysis of variance (ANOVA). *P* values were corrected using the Benjamini-Hochberg method with a false-discovery rate of 0.05.

### PNPG assay.

*p*-Nitrophenyl glucuronide (PNPG) was purchased as a solid and dissolved in water at a concentration of 100 mM. Reactions were conducted in 96-well, black, clear-bottom assay plates (Costar) at 37°C. Reaction mixtures consisted of PNPG (at various concentrations) and GUS enzyme (at various concentrations) diluted in assay buffer (50 mM HEPES and 50 mM NaCl for pH ≥6.0 or 50 mM sodium acetate and 50 mM NaCl for pH <6.0). To determine the optimal pH for *Bo*GUS L2, *Bu*GUS L2, and *Bf*GUS mini-loop 1, the assay described above was conducted at 800 μM PNPG for *Bu*GUS L2 and *Bf*GUS mini-loop 1 and 1,500 μM PNPG for *Bo*GUS in the appropriate assay buffer where the pH ranged from 4.0 to 7.4. Reactions were quenched with 0.2 M sodium carbonate, and the product formation was measured over time via absorbance at 410 nm using a PHERAstar *Plus* microplate reader (BMG Labtech). Upon determining the optimal pH for each enzyme, velocities were determined for multiple concentrations of substrate and enzyme at each enzyme’s optimal pH, and the Michaelis-Menten kinetics module in SigmaPlot 13 (Systat Software, Inc.) was used to calculate *K_m_*, *k*_cat_, and *k*_cat_/*K_m_*.

### Oligosaccharide assays.

Sulfated and nonsulfated oligosaccharide assays were performed as previously outlined (15).
