# Supplementary figures and images for: Mouse Gut Microbiome-Encoded β-Glucuronidases Identified Using Metagenome Analysis Guided by Protein Structure
Source: mSystems. 2019 Aug 27;4(4):e00452-19. doi: 10.1128/mSystems.00452-19 (PMC6712278; doi:10.1128/mSystems.00452-19)

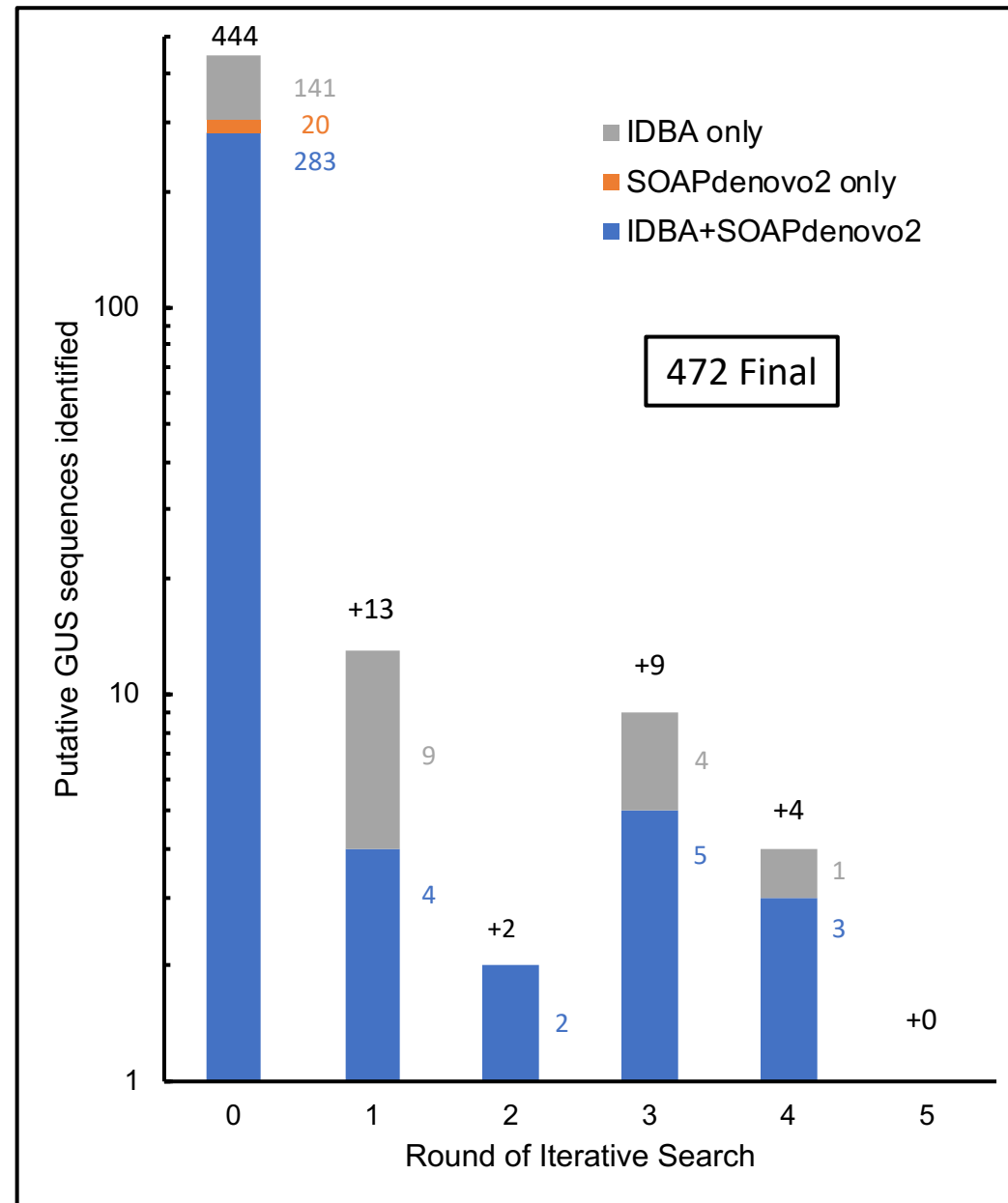

Supplement: FIG S1 [file mSystems.00452-19-sf001.pdf]

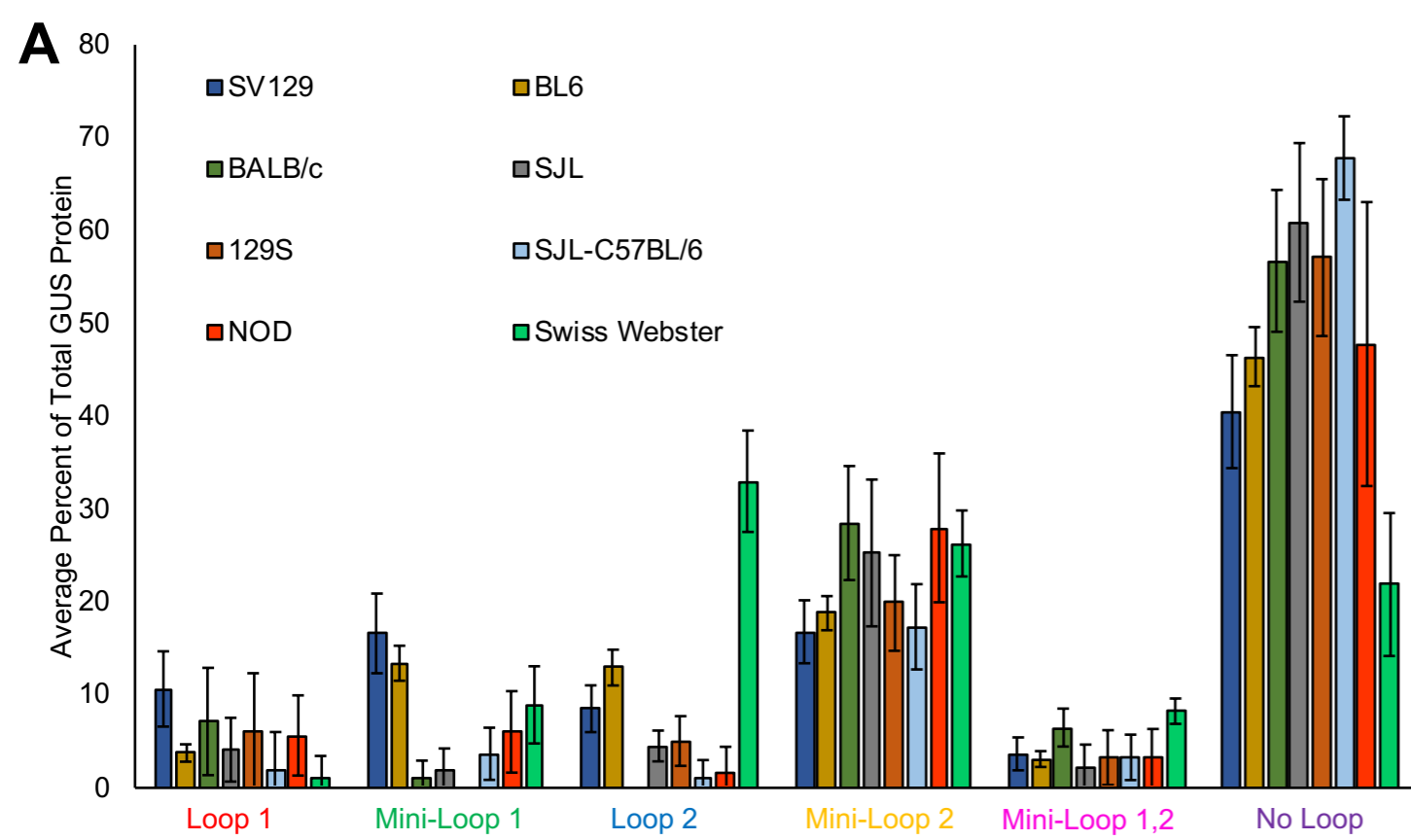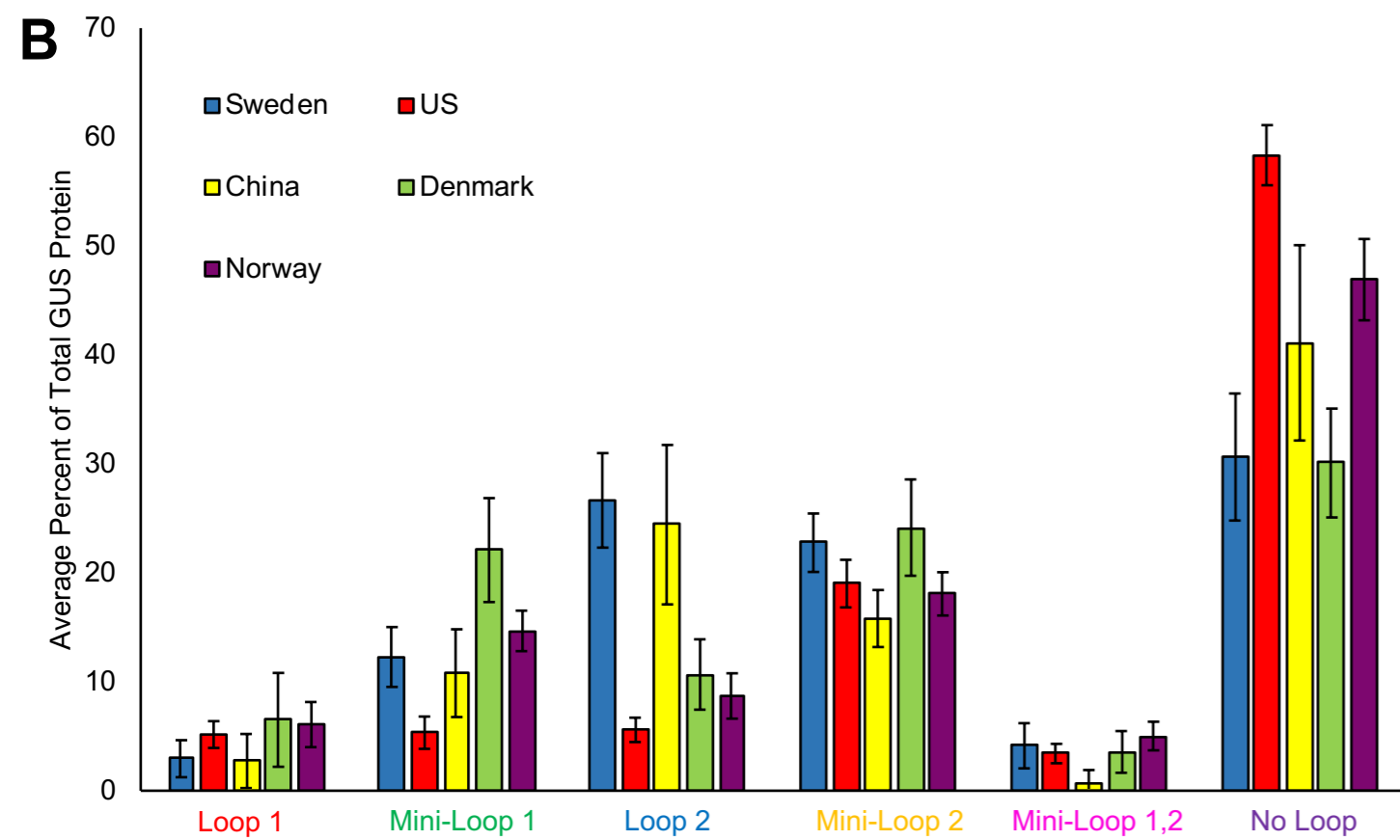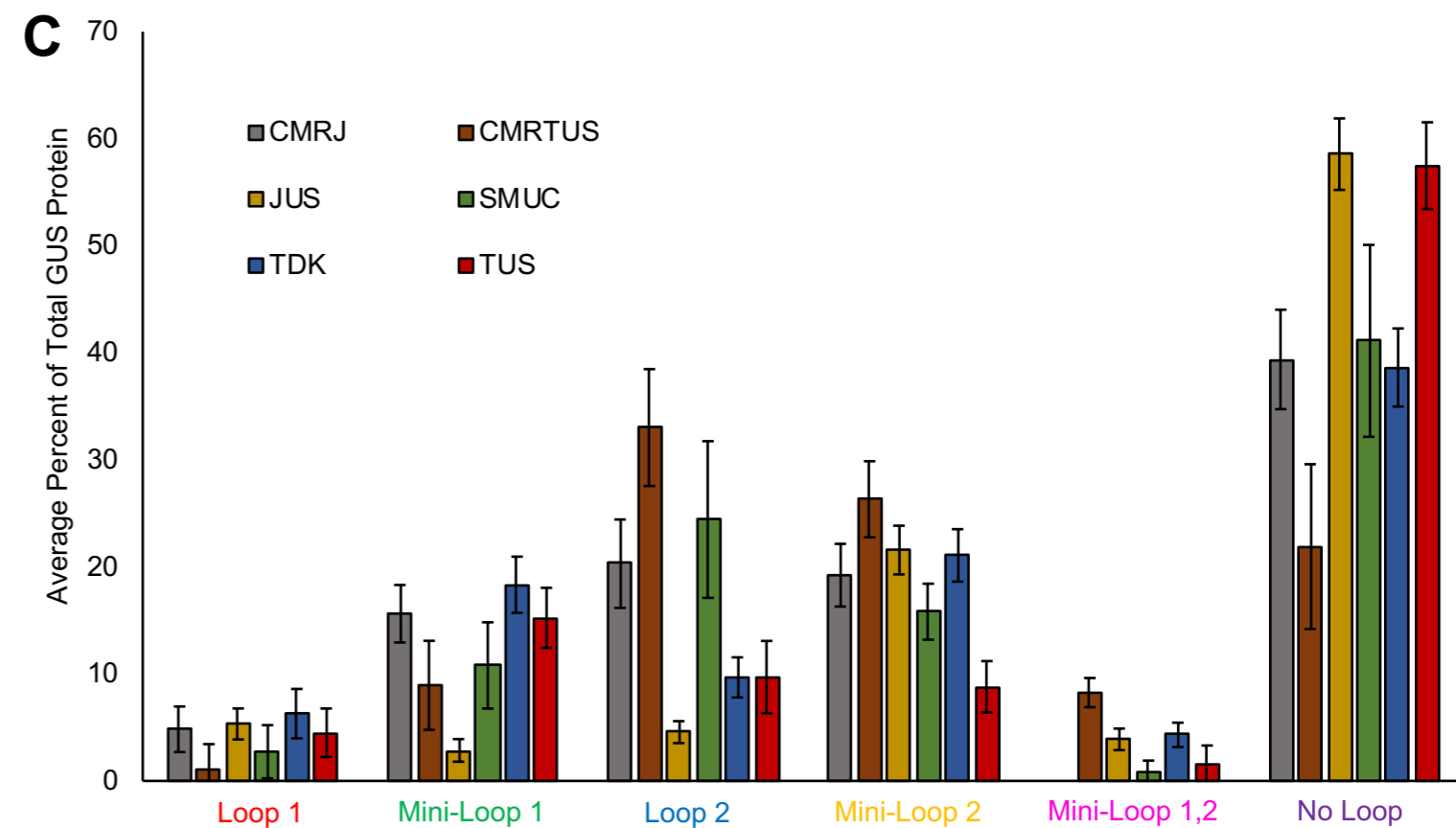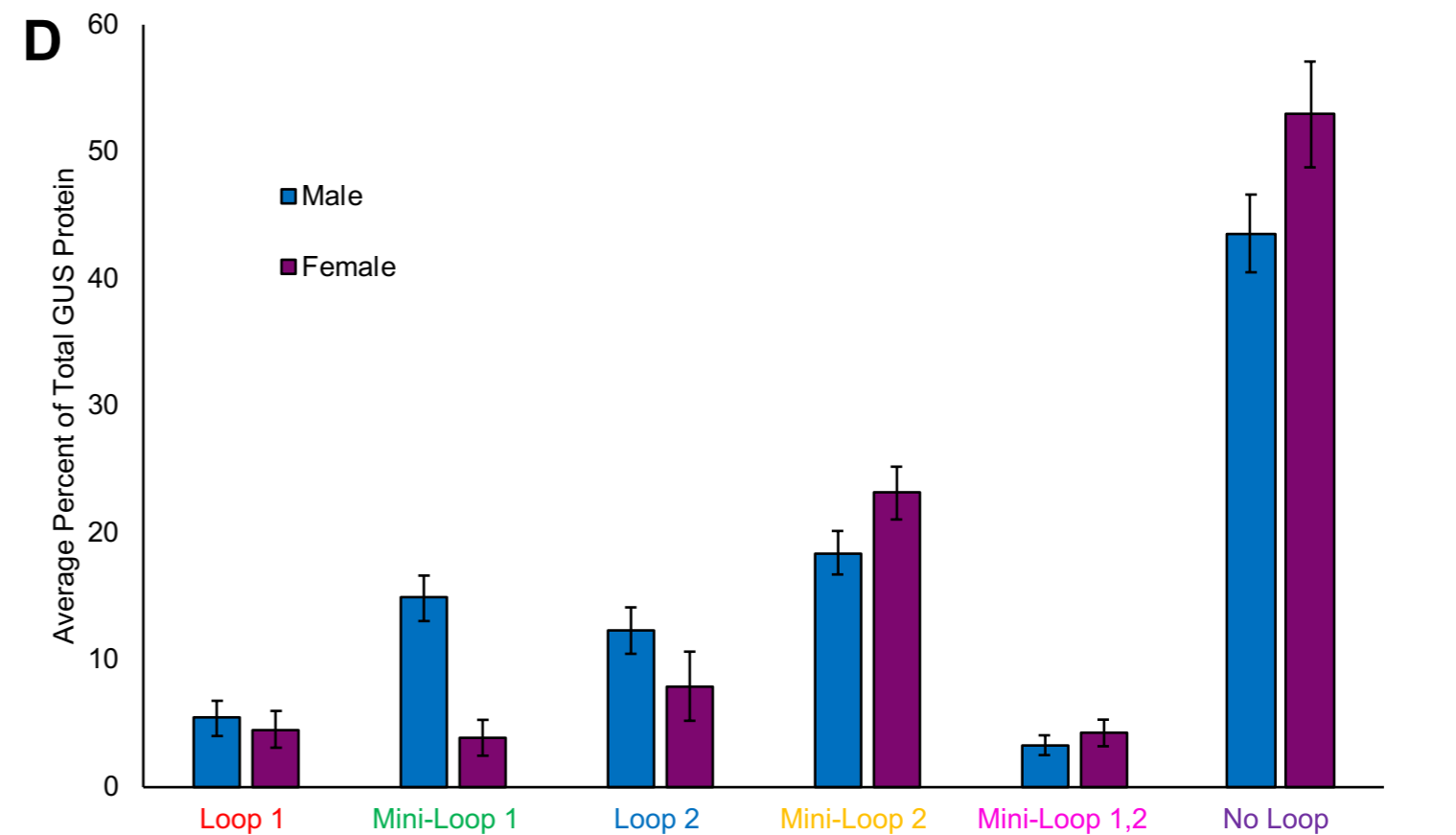

Supplement: FIG S2 [file mSystems.00452-19-sf002.pdf]

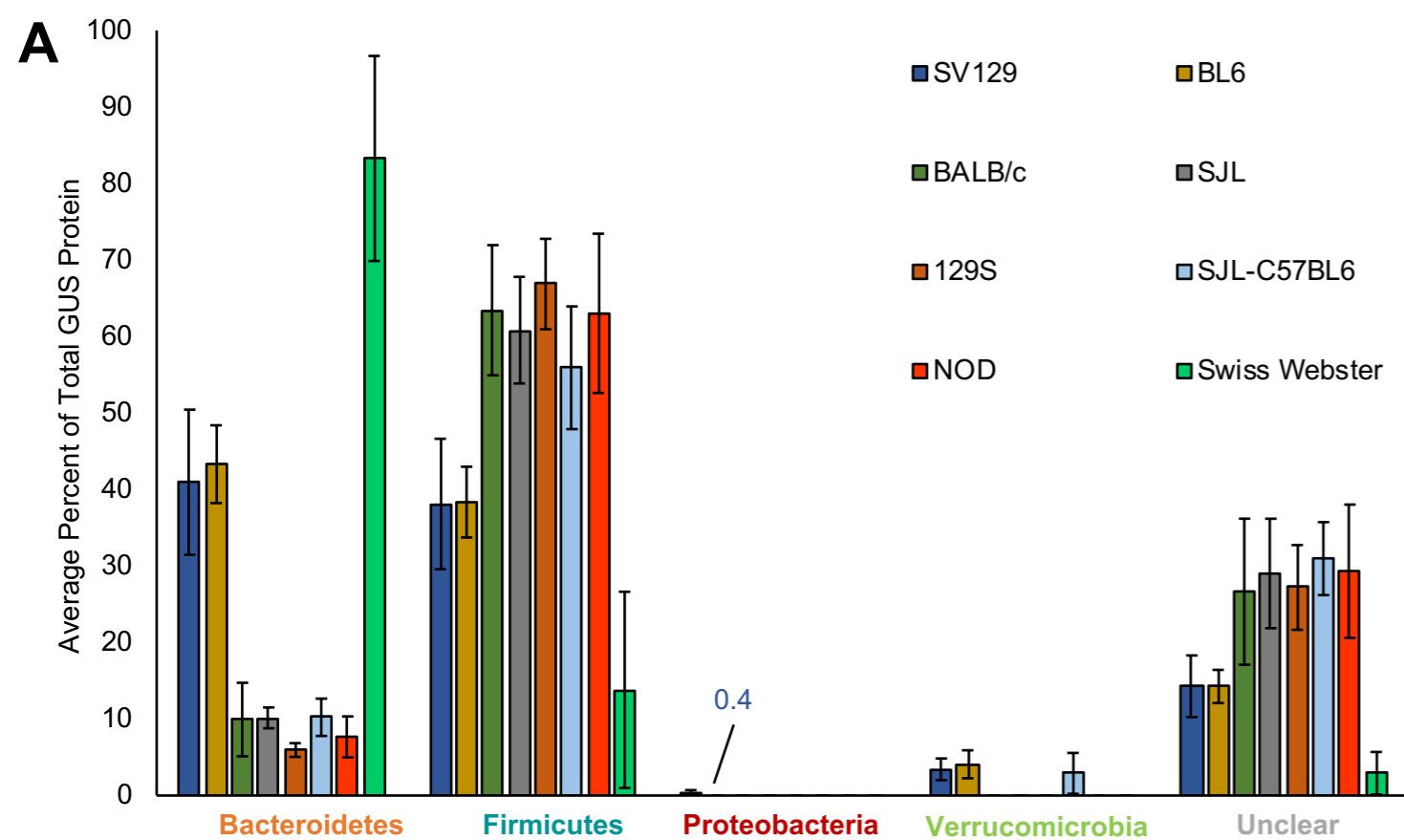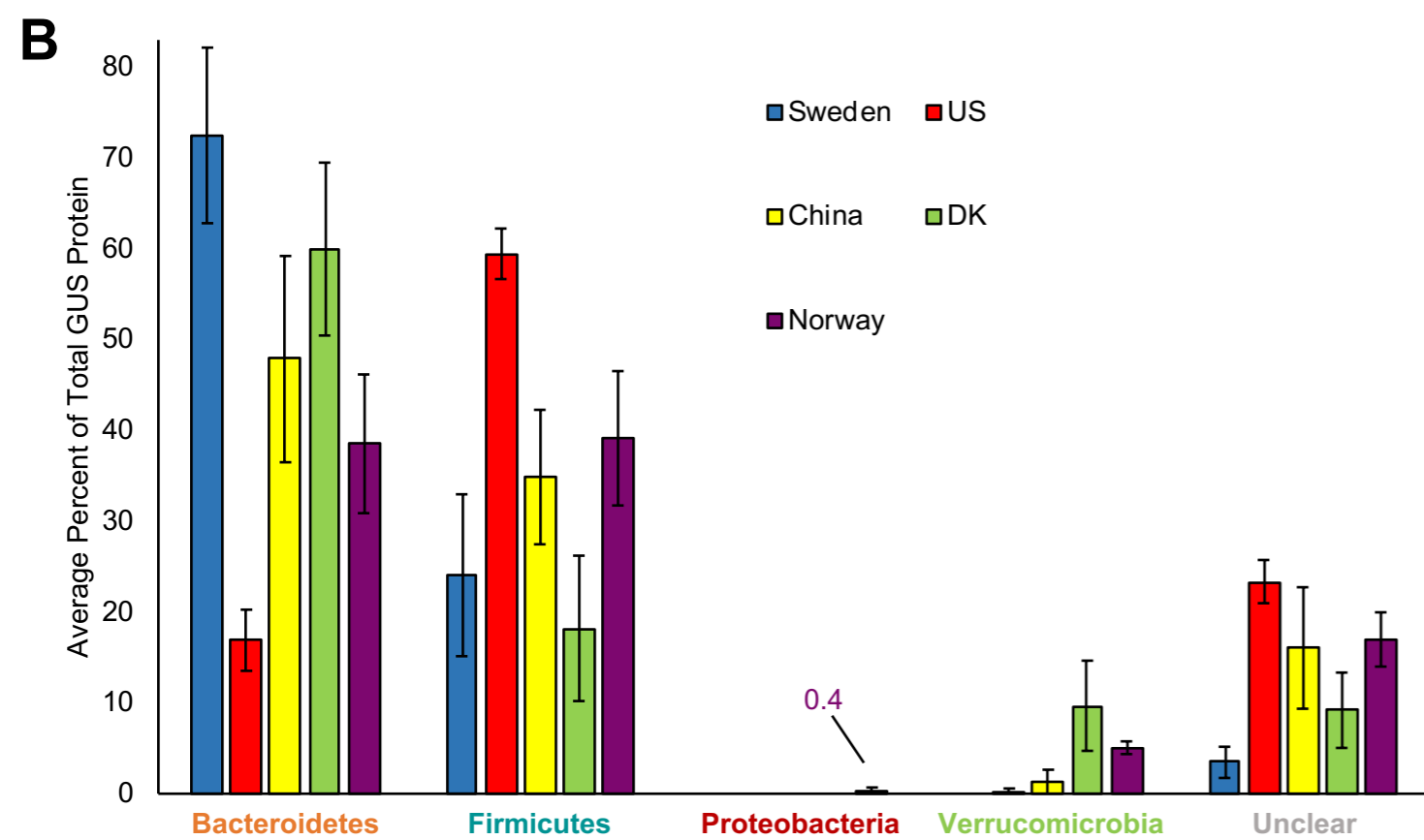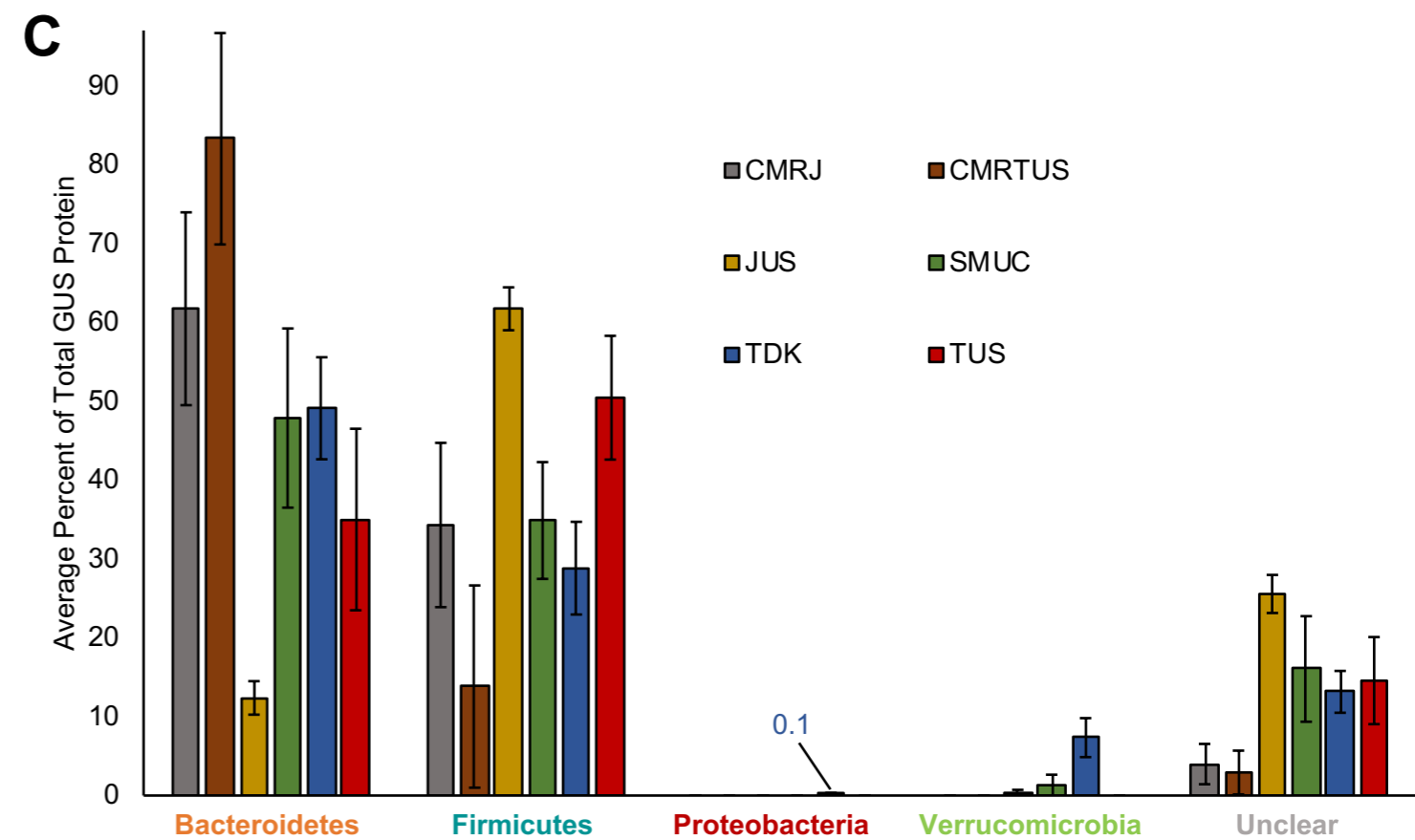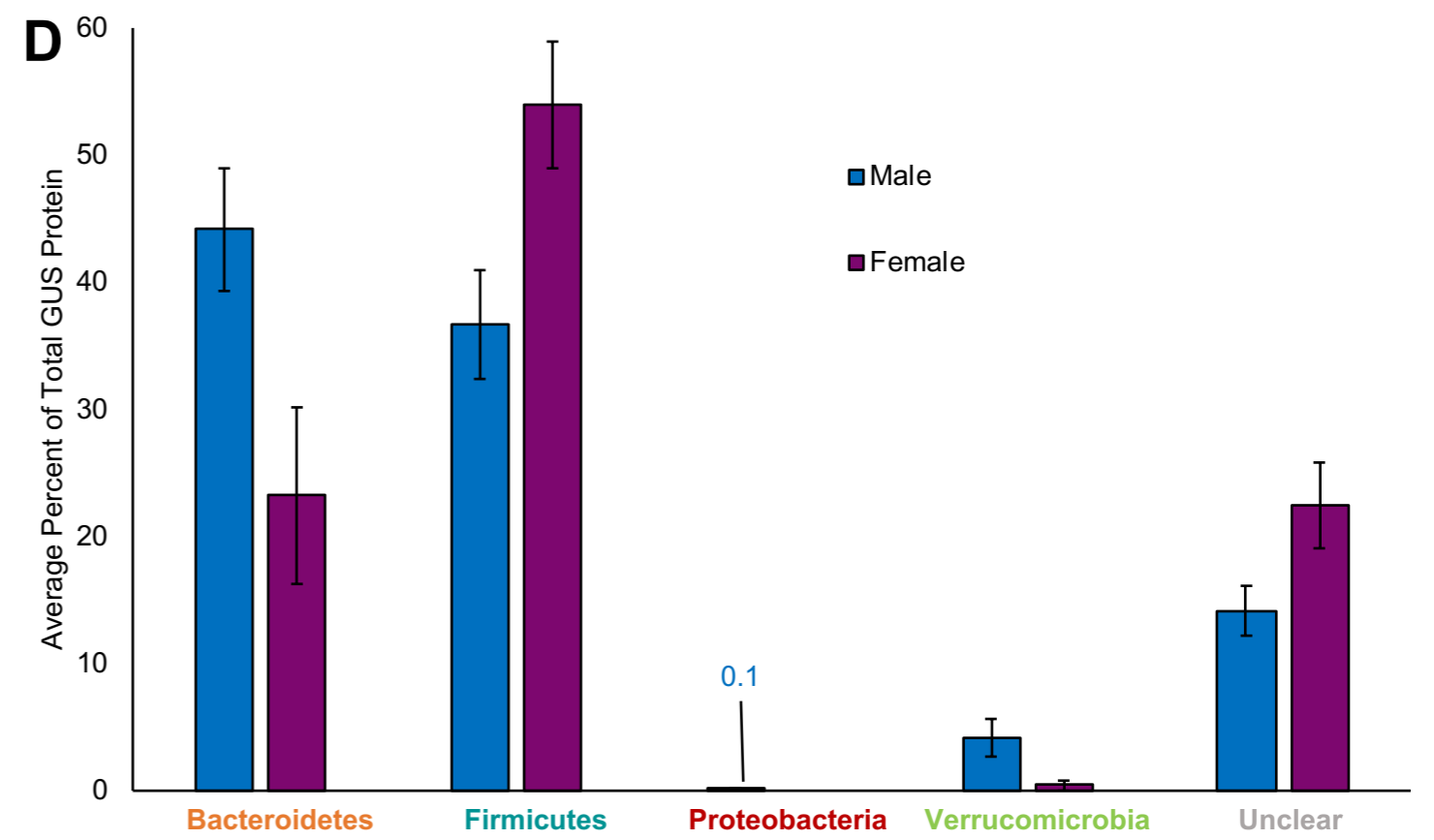

Supplement: FIG S3 [file mSystems.00452-19-sf003.pdf]

## *Bacteroides ovatus* L2 GUS

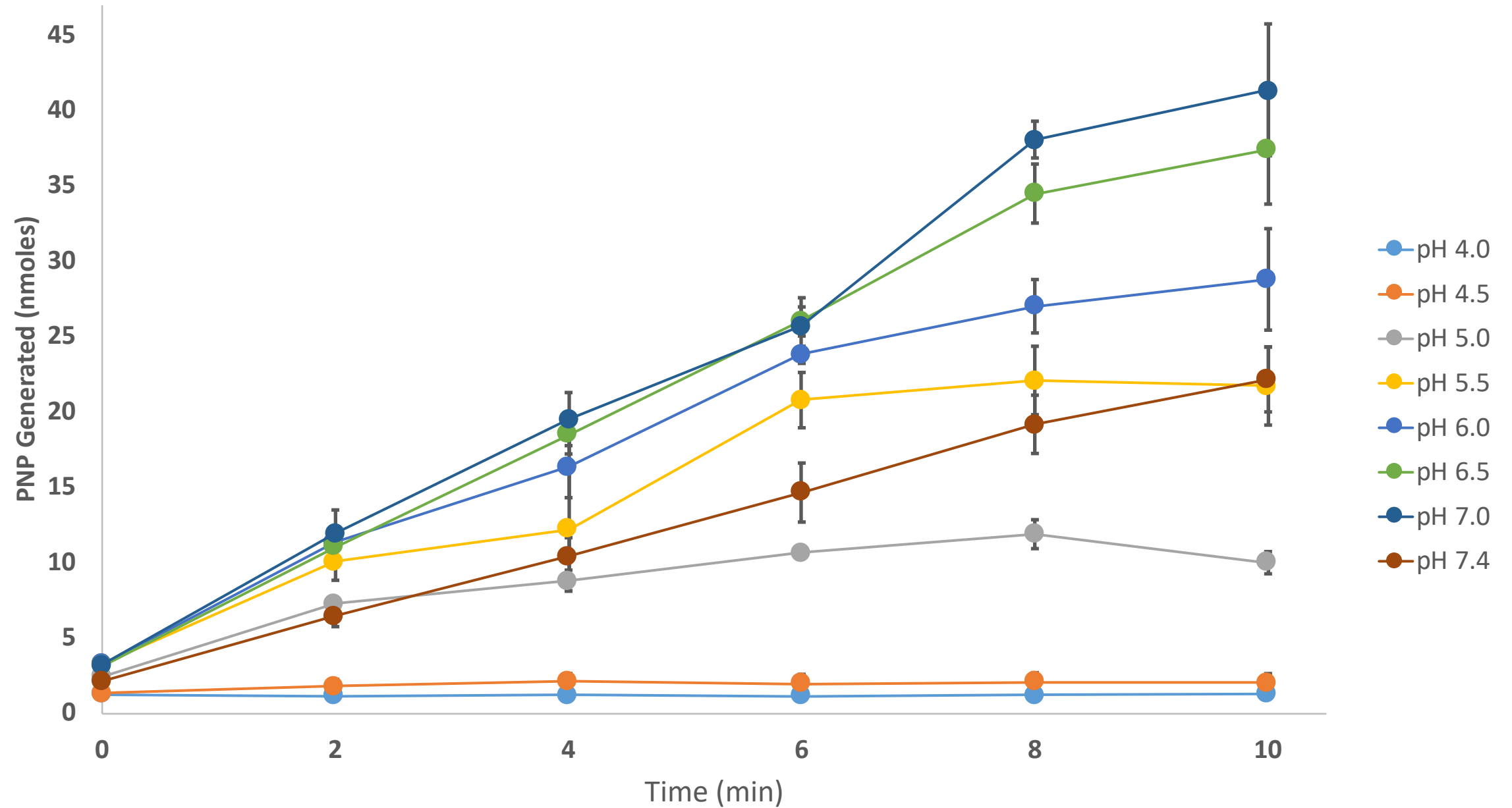

Supplement: FIG S4 [file mSystems.00452-19-sf004.pdf]

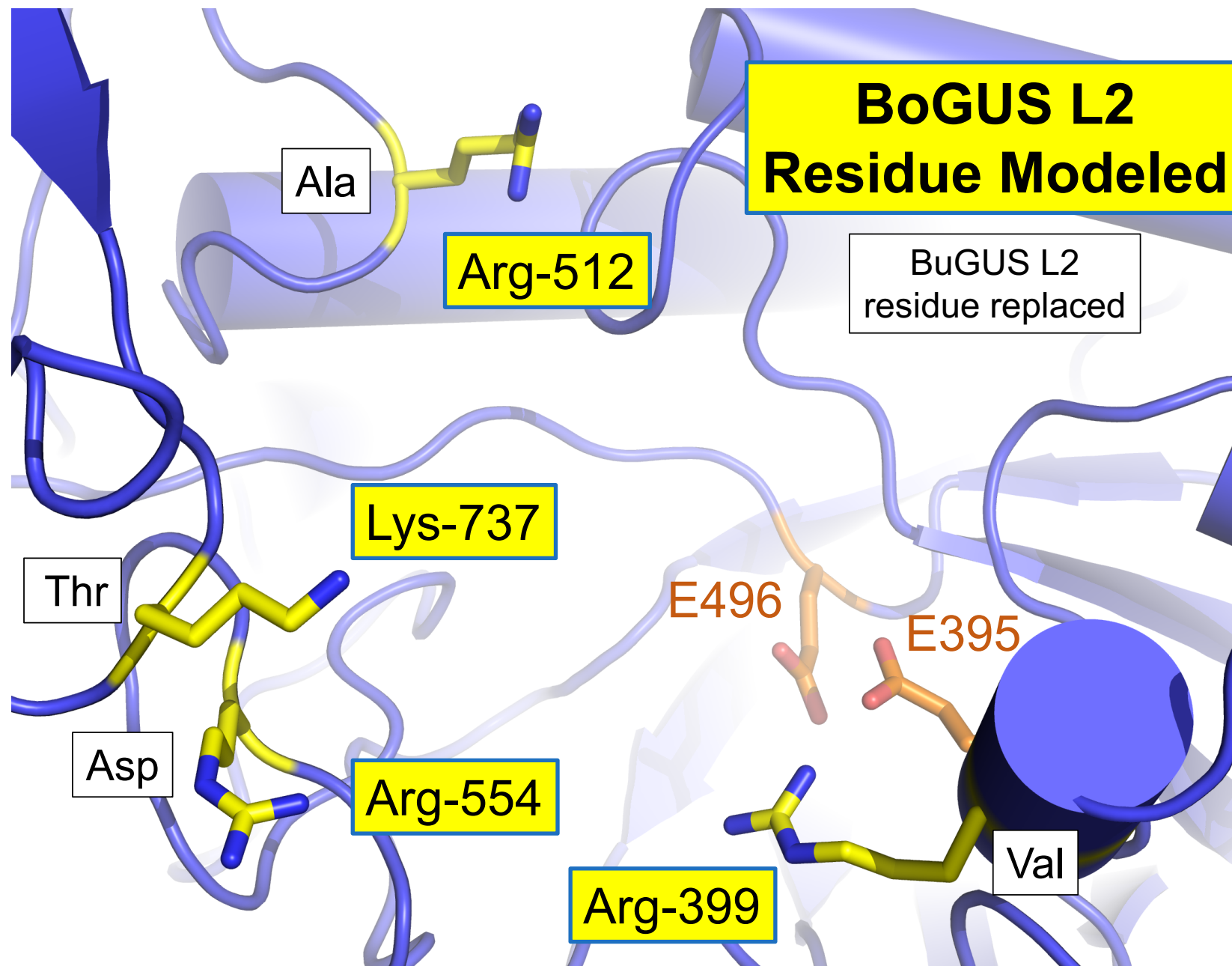

Supplement: FIG S5 [file mSystems.00452-19-sf005.pdf]
